# Supplementary material for: Effects on Child Development and Parent–Child Interaction of the FACAM Intervention: A Randomized Controlled Study of an Interdisciplinary Intervention to Support Women in Vulnerable Positions through Pregnancy and Early Motherhood
Source: Int J Environ Res Public Health. 2024 May 2;21(5):587. doi: 10.3390/ijerph21050587 (PMC11121224; doi:10.3390/ijerph21050587)
Supplement: Supplementary file 1 [file ijerph-21-00587-s001.zip › ijerph-2942295-supplementary.pdf]

Supplementary Table S1 Comparison of characteristics at baseline between families with low concern and families with medium or high concern.

	Low concern N = 240		Medium or high concern N = 91		Difference	
	Mean	SD	Mean	SD	T-stat	p-value
Mother age	30.48	(5.32)	27.42	(5.50)	4.64	0.00
Mother health	7.00	(1.81)	7.48	(1.72)	-2.18	0.03
Mother life satisfaction	7.71	(1.83)	7.79	(1.95)	-0.36	0.72
Mother well-being	23.54	(3.89)	24.33	(4.51)	-1.58	0.11
Ever lonely	2.48	(0.92)	2.54	(0.95)	-0.52	0.60
Access to practical help	3.99	(1.05)	4.21	(0.98)	-1.74	0.08
Access to somebody to talk to	4.54	(0.83)	4.53	(0.85)	0.14	0.89
HADS-anxiety	6.89	(3.75)	6.52	(4.11)	0.78	0.43
HADS-depression	4.59	(3.36)	3.89	(3.03)	1.73	0.08
PTSD total score	13.62	(5.86)	14.71	(6.25)	-1.49	0.14
ECR: Fear of abandonment	18.95	(7.12)	19.05	(6.85)	-0.12	0.90
ECR: Fear of intimacy	13.50	(6.55)	13.84	(7.11)	-0.40	0.69
PPRFQ opacity of mental states	4.62	(1.23)	4.51	(1.36)	0.68	0.50
PPRFQ reflecting on the fetus-child	4.92	(1.05)	5.40	(0.86)	-3.76	0.00
PPRFQ the dynamic nature of mental states	4.46	(1.08)	4.44	(1.14)	0.14	0.89
ACE total score	2.23	(2.10)	2.98	(2.48)	-2.63	0.01
Units of alcohol prior to pregnancy	1.52	(1.97)	1.46	(2.50)	0.24	0.81
Units of alcohol during pregnancy	0.03	(0.18)	0.00	(0.00)	1.31	0.19
Expecting first child	0.60	(0.49)	0.69	(0.46)	-1.62	0.11
Cohabit with partner	0.86	(0.35)	0.71	(0.45)	3.07	0.00
Only speak Danish at home	0.83	(0.38)	0.80	(0.40)	0.57	0.57
High school or less	0.31	(0.46)	0.69	(0.46)	-6.73	0.00
Vocational or secondary education	0.23	(0.42)	0.16	(0.37)	1.20	0.23
College, Bachelor, tertiary or longer education	0.47	(0.50)	0.14	(0.35)	5.67	0.00
Employed	0.42	(0.49)	0.21	(0.41)	3.58	0.00
Sick leave	0.20	(0.40)	0.10	(0.30)	2.11	0.04
Unemployment benefit	0.07	(0.26)	0.04	(0.21)	0.89	0.37
Social assistance/unemployment program	0.08	(0.27)	0.40	(0.49)	-7.44	0.00
In education	0.19	(0.39)	0.19	(0.39)	0.01	0.99
Unemployment no benefits	0.02	(0.13)	0.02	(0.15)	-0.32	0.75
Smoking regularly	0.08	(0.28)	0.23	(0.42)	-3.70	0.00
Never regularly used drugs like hash, pot, marihuana	0.93	(0.26)	0.75	(0.44)	4.50	0.00
Never regularly used drugs like amphetamin, ecstasy, cocaine, LSD	0.96	(0.19)	0.90	(0.30)	2.21	0.03
Used medicine during pregnancy (including non-prescriptipn painkillers)	0.63	(0.48)	0.54	(0.50)	1.58	0.12
Expect to breast feed	0.97	(0.17)	0.95	(0.22)	0.72	0.48

Supplementary Table S2 Interaction analyses with regression coefficients and p-values.

	Add. effect of mother having at more than high school education		Add. effect of mother having high level of concern		Add. Effect of mother having high trauma level	
	b	P	b	P	b	P
ASQ:SE-2 Totalscore	-7.20	0.10	-4.51	0.34	-0.17	0.97
ASQ:SE-2 Totalscore incl. worries	-4.11	0.43	3.02	0.58	-5.00	0.32
Child acitivities	-0.27	0.15	-0.24	0.26	-0.25	0.16
The Mother and Baby Interaction Scale	-0.64	0.58	0.00	1.00	0.14	0.90
CIB: Sensitivity	-0.01	0.94	0.09	0.55	0,27	0.19
CIB: Intrusiveness	-0.24	0.18	-0.10	0.55	0,09	0.73
CIB: Limit-setting	-0.26	0.08	-0.31	0.04	-0,38	0.07
CIB: Involvement	0.05	0.52	-0.25	0.06	-0,53	0.02
CIB: Withdrawal	0.21	0.01	0.10	0.51	-0,26	0.34
CIB: Compliance	0.04	0.63	0.07	0.58	0,26	0.21
CIB: Reciprocity	0.26	0.19	0.19	0.34	0,14	0.30
CIB: Negative states	-0.11	0.65	-0.28	0.22	-0,10	0.55
BSID: Cognitive scale	1.09	0.15	0.65	0.36	0.21	0.77
BSID: Receptive language scale	1.12	0.25	-0.37	0.56	-0.38	0.62
BSID: expressive language scale	1.19	0.11	0.70	0.59	0.28	0.80
BSID: Language scale	-0.69	0.39	1.33	0.31	0.58	0.45
BSID: Fine motor scale	0.78	0.35	-1.06	0.38	0.41	0.75
BSID: Gross motor scale	-0.50	0.53	0.48	0.53	0.26	0.88
BSID: Motor scale	1.15	0.11	0.14	0.88	0.68	0.58
SEAM: Empathy	-1.63	0.35	-1.51	0.44	-1.20	0.56
SEAM: Selfregulation and positive self-image	-0.23	0.78	-1.08	0.32	-0.24	0.82
Observations	Survey outcomes N=324, CIB N = 194, BSID N = 196, SEAM N = 164					

Supplementary Table S3 Sensitivity analyses.

	Complete case				Instrumental variable analysis			
	T2 child age 3-6 months		T3 child age 12-14 months		T2 child age 3-6 months		T3 child age 12-14 months	
	b	P	b	P	b	P	b	P
Child health	-0.07	0.63			-0.08	0.62		
ASQ:3 Communication	-0.28	0.81			-0.32	0.81		
ASQ:3 Gross motor	-0.10	0.93			-0.11	0.93		
ASQ:3 Fine motor	1.29	0.47			1.44	0.46		
ASQ:3 Problem solving	-0.18	0.90			-0.21	0.90		
ASQ:3 Personal-social	-0.69	0.63			-0.77	0.63		
ASQ:SE Totalscore	-0.10	0.96	-0.54	0.80	-0.12	0.96	-0.61	0.80
ASQ:SE Totalscore incl. worries	0.64	0.84	0.25	0.92	0.71	0.84	0.29	0.92
Child acitivities			0.08	0.40			0.09	0.39
The Mother and Baby Interaction Scale			1.05	0.05			1.18	0.05
CIB: Sensitivity					-0.13	0.15	-0.07	0.37
CIB: Intrusiveness					0.04	0.53	0.04	0.26
CIB: Limit-setting					-0.06	0.60	-0.16	0.13

CIB: Involvement			-0.27	0.00	-0.09	0.24
CIB: Withdrawal			0.16	0.06	0.07	0.28
CIB: Compliance					-0.11	0.30
CIB: Reciprocity			-0.17	0.14	-0.11	0.32
CIB: Negative states			0.20	0.07	0.13	0.23
BSID: Cognitive scale					-0.47	0.22
BSID: Receptive language scale					-0.38	0.35
BSID: expressive language scale					-0.12	0.72
BSID: Language scale					-0.41	0.51
BSID: Fine motor scale					0.69	0.07
BSID: Gross motor scale					0.38	0.36
BSID: Motor scale					1.21	0.07
SEAM: Empathy					-0.34	0.83
SEAM: Selfregulation and positive self-image					-0.05	0.94
Observations	N =284	N=248	Survey N = 284, CIB=189		Survey N = 248, CIB=194, BSID = 196, SEAM = 164	
